# Chitosan-Encapsulated Nano-selenium Targeting TCF7L2, PPARγ, and CAPN10 Genes in Diabetic Rats

**DOI:** 10.1007/s12011-022-03140-7

**Published:** 2022-03-02

**Authors:** Omayma A. R. Abozaid, Sawsan M. El-Sonbaty, Neama M. A. Hamam, Moustafa A. Farrag, Ahmad S. Kodous

**Affiliations:** 1 Clinical Biochemistry Department, Faculty of Veterinary Medicine, Benha University, Moshtohor, Egypt; 2 Radiation Microbiology Department, National Center for Radiation Research and Technology, Egyptian Atomic Energy Authority, Cairo, Egypt; 3 Radiation Biology Department, National Center for Radiation Research and Technology, Egyptian Atomic Energy Authority, Cairo, Egypt

**Keywords:** Diabetes mellitus, Selenium nanoparticles, Chitosan, Glibenclamide, TCF7L2, CAPN10, PPAR-γ

## Abstract

This study investigates the antidiabetic and antioxidant potential of chitosan-encapsulated selenium nanoparticles in streptozotocin-induced diabetic model. Glibenclamide was used as a reference antidiabetic drug. Forty-eight adult male Wistar rats were used along the study and divided equally into 6 groups of (I) normal control, (II) chitosan-encapsulated selenium nanoparticles (CTS-SeNPs), (III) glibenclamide, (IV) streptozotocin (STZ), (V) STZ + CTS-SeNPs, and (VI) STZ + Glib. The animals were sacrificed on the 35th day of the experiment. Serum glucose, insulin, IGF-1, ALT, AST, CK-MB, oxidative stress, lipid profile, and inflammatory parameters were subsequently assessed. Also, the expression level of TCF7L2, CAPN10, and PPAR-γ genes were evaluated using qPCR. In addition, histopathological studies on pancreatic tissue were carried out. The results revealed that STZ induced both diabetes and oxidative stress in normal rats, manifested by the significant changes in the studied parameters and in the physical structure of pancreatic tissue. Oral administration of CTS-SeNPs or Glib results in a significant amelioration of the levels of serum fasting blood glucose, insulin, IGF-1, AST, ATL, and CK-MB as compared with STZ-induced diabetic rats. CTS-SeNPs and Glib diminished the level of lipid peroxidation, increased total antioxidant capacity level, as well as possessed strong inhibition against serum α-amylase and α-glucosidase activities. Diabetic animals received CTS-SeNPs, or Glib demonstrated a significant (*p* < 0.05) decrease in the expression level of TCF7L2 and CAPN10 genes with a significant increase in the expression level of PPAR-γ gene, compared to STZ group. The above findings clarify the promising antidiabetic and antioxidant effect of CTS-SeNPs, recommending its inclusion in the currently used protocols for the treatment of diabetes and in the prevention of its related complications.

**Graphical abstract:**

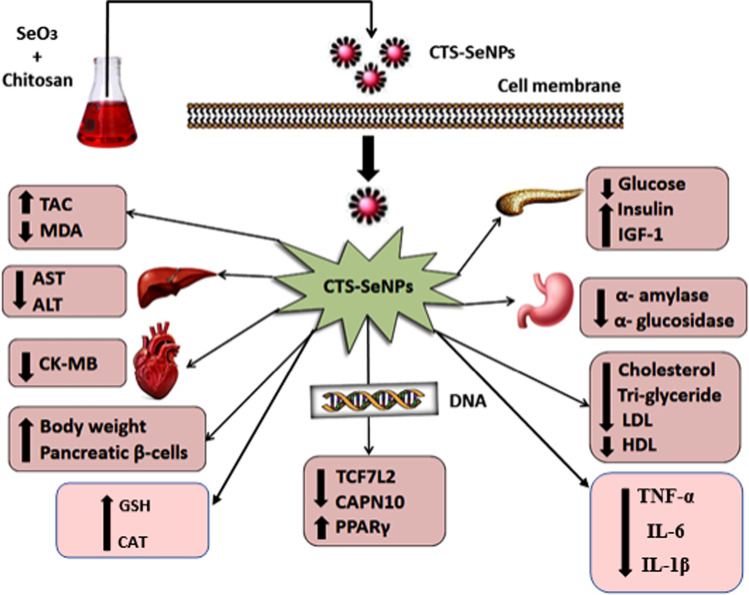

## Introduction

Diabetes mellitus (DM) is a growing epidemic that affects quality of life. It is one of the leading causes of morbidity and mortality throughout the world [1]. Diabetes mellitus (DM) is a metabolic disease characterized by chronic hyperglycemia, as well as glucose, adipose tissue, and protein metabolism disorders caused by impaired insulin production by pancreatic β-cells and/or insulin resistance by peripheral tissues [2]. In 2019, about 9.3% of adults aged 20–79 are living with DM in the Middle East and North Africa Region. Egypt is the nation with the ninth-largest population of diabetics in the world. According to the International Diabetes Federation (IDF), there were 8.2 million (14.5%) diabetic patients in Egypt in 2017. It is expected that this number will bounce up to 13.1 million by 2035 [3–5]. Diabetes mellitus is associated with several macro-vascular and microvascular complications that ultimately impact the overall patient’s survival. It has been reported that oxidative stress is a known pathway in the pathogenesis of diabetic complications [6]. Hyperglycemic-induced oxidative stress is thought to enhance proinflammatory protein levels, with infiltrated macrophages secreting inflammatory cytokines, resulting in local and systemic inflammation [7]. Furthermore, several studies have found elevated levels of IL6 and TNF-α in patients with insulin resistance and clinically diagnosed diabetes. Chronic hyperglycemia is an important factor that may contribute to inflammation [8]. Alongside with oxidative stress and hyperglycemia, hyperlipidemia has been prescribed as a causative factor for augmenting morbidity and mortality in diabetic individuals [9].

Finding efficient multifunctional therapeutic agents that act more broadly to repress diabetic complications may offer a greater advantage. One of the most promising tools in DM management is the use of natural health products. Several trace metals, including chromium, selenium, vanadium, molybdenum, and magnesium, have been known to have hypoglycemic activity, possibly due to insulin-mimetic effects [10, 11]. Thus, dietary supplementation with well-studied trace metals could be a promising treatment option for DM patients, acting in addition to approved pharmacological therapies. Currently, several nanoparticles are used as alternative therapies due to their multifunctional biological activities, which treat diabetic complications and combat inflammation. Interestingly, selenium is an essential antioxidant and anti-inflammatory micronutrient that can prevent ROS production by improving the activity of GPx and selenoproteins [12]. Additionally, Se-NPs have unique biological advantages with excellent bioavailability and a sevenfold lower toxicity than sodium selenite [13]. Besides its unique abilities, for more stability of Se-NPs in solutions, stabilizing agents such as polysaccharides are added; the active hydroxyl groups in polysaccharides can improve the bioavailability and biological activity, producing a synergistic effect between Se-NPs and polysaccharides [14].

Chitosan is a naturally occurring, linear polysaccharide that is regarded as the second most important renewable biomaterial after cellulose in terms of utilization and distribution [15].

Due to their unique biological properties, chitosan and its derivative biomaterials have piqued the interest of researchers in the biomedical field. Chitosan’s non-toxicity, biodegradability, biocompatibility, and immunoenhancing, antitumoral, antibacterial, and antimicrobial activity are among its most notable medical properties [16]. Chitosan-based nanoparticles with high permeability and retention can also inhibit tumor cell growth by inducing apoptosis. Particles as small as 100–200 nm can be taken up by receptor-mediated endocytosis, whereas larger particles must be taken up by phagocytosis [17].

Hence, the objective of this study is to investigate the antidiabetic potential of chitosan-encapsulated selenium nanoparticles as well as explore its antioxidant and cardioprotective activity in streptozotocin-induced diabetic rats.

## Materials and Methods

### Chemicals

Streptozotocin (STZ), sodium selenite (Na_2_SeO_3_), acetic acid and chitosan-extracted from shrimp shells were purchased from Sigma Chemical, St. Louis, USA. The commercial pharmaceutical drug glibenclamide (Glib) is of dietary supplement tablets (Dianil® 5 mg, Sanofi, Egypt).

### Synthesis Chitosan-Selenium Nanoparticles (CTS-SeNPs)

Biosynthesis of CTS-SeNPs was processed by a method of Hien et al. [15]. CTS solution (1% w/v) was dissolved in a diluted acetic acid solution (3% w/v). After dissolution, CS solution was stored in the dark (24.0 ± 2 °C) overnight to ensure the homogeneity. Additionally, 1 mM of selenium dioxide solution was mixed with CS solution (v/v). Finally, the mixture was stirred at room temperature for 20 min and then exposed to a dose of 20 kGy, with the dose rate of 2.5 kGy/h. The formation of Se NPs was indicted by the appearance of red-colored solution.

### Characterization of CTS-SeNPs

#### Transmission Electron Microscopy (TEM)

Synthesized CTS-SeNPs was characterized by using transmission electron microscope (TEM) of JOEL JEM-2100, microscope with an accelerating voltage of 200 kV, attached to Gatan Digital Camera, Model Erlangshen ES500.

#### Ultraviolet–Visible Absorption

Spectrum absorbance of CTs-Se NPs was scanned at range of 200–600 nm by ultraviolet–visible (UV–VIS) spectrophotometer using Jenway UV spectrophotometer model 6505.

#### Dynamic Light Scattering (DLS)

Sample of CTS-SeNPs was analyzed for size dimensions by DLS Zetasizer (ZS) which was manufactured in Malvern, UK.

#### Acute Toxicity Study

Acute oral toxicity study was performed according to the 423 guidelines (acute toxicity class method) lay down by OECD (Organisation of Economic Cooperation and Development). Healthy male Wistar rats were randomly divided into ten groups with five animals in each group.

A single acute dose of CTS-SeNPs prepared in sterile distilled water was administered orally by gavage at five different concentrations (2, 5, 7.5, 10, and 15 mg/kg Se). Toxicity signs and mortality were observed within 7 days after the treatment. Interestingly, oral administration of SeNPs did not show mortality at any of the concentrations tested.

The arithmetic method of Karber [18] was used for the determination of LD50. LD50 = LD100 − ∑ (*a* × *b*)/*n*. *n* is the total number of animal in a group, *a* the difference between two successive doses of administered extract/substance, *b* the average number of dead animals in two successive doses, and *LD100* lethal dose causing the 100% death of all test animals.

#### Induction of Diabetes

Rats were given free access to water and either a standard pellet diet (APRI animal feed) from Animal Production Research Institute (APRI), Dokki, Giza, Egypt, with carbohydrate, protein, and lipids by 72.1, 22.1, and 5.7%, respectively, and a total caloric value of 3100 kcal/kg, or a high-fat diet (HFD) with carbohydrate, protein, and lipids by 27.5, 14.5, and 58.8%, respectively, and a total caloric value of 4900 kcal/kg. As according to the American Institute of Nutrition [19], corn oil and beef tallow were the fat source in the diet, as well as butter and casein as a protein source in the diet since casein has an acceptable amino acid composition. Casein’s main constraint is a lack of sulfur amino acids, notably cystine/cysteine. This deficiency was corrected by supplementing the diet with DL-methionine, which is turned to cysteine in the body [19]. Table 1 shows the composition of the HFD.

**Table 1:** Composition of high-fat diet

Ingredients	Diet (g/kg)
Normal pellet diet	585.4
Butter	310.9
Casein	73.2
Mineral mix	24.6
Vitamin mix	4.1
dl-methionine	1.8

Wistar rats were received HFD for 2 weeks followed by single intraperitoneal injection of STZ dissolved in 0.1 M freshly prepared sodium citrate buffer (pH = 6.5) at a dose of (50 mg/kg b.wt) [20].

Seventy-two hours after induction, blood samples were obtained from the tips of the rat’s tail, and the fasting blood glucose levels were determined using OneTouch Ultra glucometer (LifeScan, USA) to confirm diabetes. Rats with fasting blood glucose levels of ≥ 200 mg/dL were used for the experiment [16].

#### Experimental Design

**Fig. 1:**
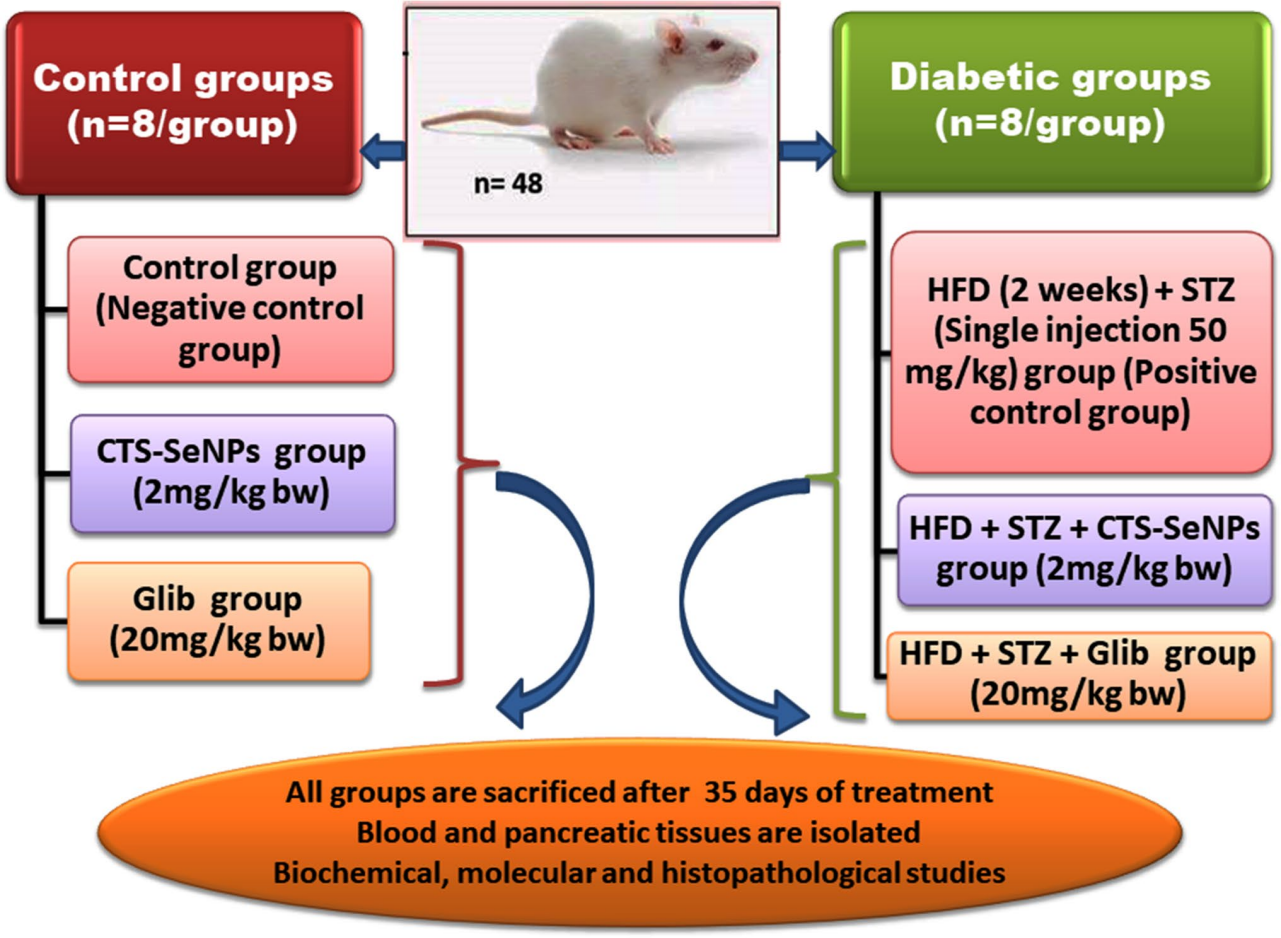
Experimental design. Group (normal control), Group II (CTS-SeNPs): rats were administered with CTS-SeNPs [17]. Group III (Glib): nondiabetic rats were administered with Glib [17]. Group IV **(**STZ) (diabetic control). Group V (STZ + CTS-SeNPs). Group VI **(**STZ + Glib)

Forty-eight male Wistar rats were obtained from the Nile Pharmaceutical Co., Cairo, Egypt, with body weight 150–200 gm. Rats were housed at the animal house of the National Center for Radiation Research and Technology (NCRRT) and the Egyptian Atomic Energy Authority (EAEA), Cairo, Egypt. Rats were randomly divided into 6 groups of 8 animals in each group (Fig. 1).

Body weights were recorded along the experimental period at 7, 14, 21, 28, and 35 days of the experimental time. After 35 days, the animals were fasted for 12 h, anaesthetized using ketamine (24 mg/kg b.wt. intramuscular injection), and sacrificed by decapitation. Blood samples for serum were collected. Additionally, pancreas was excised and divided into two parts as follows: 1st was used for the determination of gene expression levels of TC7L2, PPAR-γ, and CAPN10 by real-time PCR. The 2nd was fixed in 10% neutral formalin overnight, embedded in paraffin, and cut into 4–5-µm sections for histological analysis following hematoxylin and eosin staining.

### Biochemical Analyses

#### Determination of Fasting Blood Glucose

Blood glucose level was measured via the glucose oxidase method according to Trinder [21] using a glucose assay kit of Spectrum-Diagnostics, Cairo, Egypt.

#### Determination of Serum Insulin, TNF-α, IL-6, IGF-1, and IL-1β

The serum insulin, TNF-α, IL-6, IGF-1, and IL-1β concentrations were measured by an enzyme-linked immunosorbent assay method using kits from MYBiosource Company according to the manufacturer’s instructions.

#### Determination of the Oxidant and Antioxidant Parameters

The activity of catalase (CAT) and reduced glutathione (GSH) were determined as described in commercial kits (Randox Laboratories Ltd, Antrim, UK). Total antioxidant capacity (TAC) and malondialdehyde (MDA) concentrations were determined as described in commercial kits (Biodiagnostic Co., Egypt).

#### Determination of Hepatic and Cardiac Enzymes

Serum hepatic enzymes alanine transaminase (ALT), aspartate transaminase (AST), alkaline phosphatase (ALP), gamma-glutamyl transferase (GGT), total bilirubin, and albumin were measured by kit methods (Biodiagnostic Co., Egypt). Moreover, serum CK-MB fraction activity was detected using kit of BIOMED diagnostic according to Wu and Bowers [22].

#### Determination of α-Amylase and α-Glucosidase Activity in Serum

The activity of both α-amylase and α-glucosidase was determined in serum spectrophotometrically using kits of Biodiagnostic Co., Egypt, and Biovision, Egypt, respectively.

#### Determination of Lipid Profile

The relative clinical biochemical parameters such as TG, HDL-C, and LDL-C were measured using available diagnostic kits (Biodiagnostic Co., Egypt) following the respective kit specifications.

#### Molecular Analysis

Total RNA isolation from pancreatic tissues was performed using RNeasy Plus Minikit (Qiagen, USA) to measure the expression level of the specified genes. cDNA was synthesized by following the protocol provided with the iScript™ cDNA synthesis kit (Bio-Rad, CA). Real-time PCR was carried out via Power SYBR® Green (Life Technologies, CA) and the Applied Biosystems, 7500 Instrument. The reference gene used was β-actin, and cDNA samples were run in triplicates. The thermal cycling program used was as follows: heating at 95 °C for 10 min during the initial denaturation stage, 95 °C in the amplification phase for 10 s which was left to run 40–45 cycles, 66 °C for 10 s in the annealing phase, and finally 72 °C for 20 s in the extension phase. The single fluorescence was captured for each capillary to perform signal detection. The cycle threshold (CT) was measured for both target genes. Subsequently, the relative quantization (RQ) for each sample was calculated using a specific formula to normalize the expression towards the housekeeping gene also to be able to compare it with the control. At first, the Δ CT was measured for each sample, in which ^Δ^CT = CT related to the target gene – CT of the housekeeping gene. Secondly, the ^ΔΔ^CT was evaluated for each sample in which ^ΔΔ^CT = ^Δ^CT related to the experimental sample and – ^Δ^CT is for the control. Finally, the RQ was calculated where RQ = 2^− ΔΔCT^.

The PCR primers for gene were designed using the NCBI, Primer-Blast program, which were fabricated using Euro fins MWG Operon (Huntsville, AL). Their sequence is indicated in Table 2.

**Table 2:** Primer sequence of CAPN10, TCF7, PPAR–γ, and β-actin

Gene symbol	Primer sequence
CAPN10	Forward: 5′- AACCCAGCGAGGTGTGTGTGGCTGTT-3′ Reverse: 5′GCAGTGTTGCTGTAGGGTGATACGGATG-3′
TCF7	forward: 5′-GAG TGC GAA ATC CCC AGT TA-3′ reverse: 5′-ATG CAT GGC TTC TTG CTC TT-3′
PPAR-γ	Forward: 5′-AAGCCATCTTCACGATGCTG-3′ Reverse: 5′-TCAGAGGTCCCTGAACAGTG-3′
β-actin	Forward: 5′-TGTTGTCCCTGTATGCCTCT-3′ Reverse: 3′-TAATGTCACGCACGATTTCC-5′

#### Histopathological Study

Specimens from pancreas tissue were fixed in 10% buffered neutral formalin solution, dehydrated, embedded in bee wax paraffin, and then cut to 5-μm-thick paraffin sections on glass slides. Specimens were stained with hematoxylin and eosin “H&E” by routine procedure of Banchroft et al. [23].

#### Statistical Analysis

Statistical analyses were performed using IBM, SPSS program (version 22Inc, Chicago, USA). Differences between groups were calculated using one-way analysis of variance (ANOVA) followed by Duncan’s test post hoc analysis. All values were expressed as Mean ± SD, and differences were considered significant at *P* < 0.05.

## Results

### Characterization of the Synthesized CTS-SeNPs

**Fig. 2:**
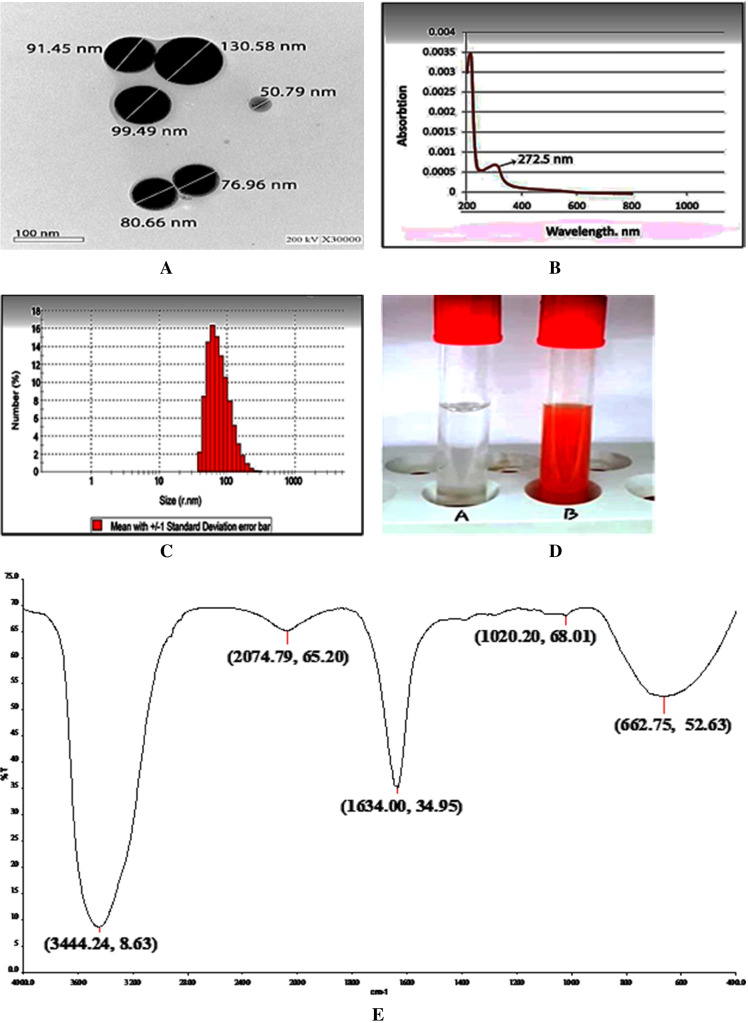
TEM image with scale bar = 100 nm, b absorption spectrum by scanning spectrophotometer, c histogram of DLS analysis for nanoparticles size distribution, d photo of CTS-SeNPs samples: A before nanoparticle formation, B after nanoparticle formation developing a reddish color, and e Fourier transform infrared spectra for CTS-SeNPs

Identification of specific characters of the prepared CTS-SeNPs such as particle shape and size was investigated via TEM analysis. CTS-SeNPs showed well-identified spherical shape, with a size around 50–130 nm as shown in Fig. 2a. The formation of SeNPs was confirmed by changing the color of the solution into red and detection of a max-peak in the visible region at 272.5 nm characteristic for CTS-SeNPs (Fig. 2b and d). DLS results showed that CTS-SeNPs size is ranged from 39.4 to 265.6 nm with high percentage of sizes 52.85, 61.2, 82.09, and 95.07 of 14.5, 16.4, 12.9, and 10.6% respectively (Fig. 2c). FTIR spectrum of CTS-SeNPs (Fig. 2e) shows a strong signal at 3444.24 representing –OH, at 1634.00 representing amid I of proteins, and at 662.75 representing carbon with –H or –N which with other weak peak bonds named finger print.

### Determination of Acute Toxicity Median Lethal Dose (LD_50_) of CTS-SeNPs in Rats

Oral administration of different concentrations of the synthesized CTS-SeNPs ranging 2–15 mg/Se/kg. b.wt did not produce any mortality.

### Effect of STZ, CTS-SeNPs, or Glib on Serum Glucose, Insulin, and IGF-1 Levels in Diabetic Rats

Intraperitoneal injection of STZ to normal animals induced a marked increase in serum glucose level, confirming the diabetogenic effect of STZ, whereas the levels of serum insulin and IGF-1 were significantly decreased (1.4 ± 0.19 and 11.5 ± 3.5, respectively) as compared to control group. Remarkably, administration of CTS-SeNPs or Glib to diabetic rats significantly (*p* ≤ 0.05) reduced glucose levels relevant to STZ group. Simultaneously, treating diabetic rats with CTS-SeNPs or Glib significantly (*p* ≤ 0.05) restored the levels of serum insulin and IGF-1 as compared to STZ group. On the other hand, no significant changes were observed in the level of these parameters between normal controls, CTS-SeNPs, or Glib animals (Table 3).

**Table 3:** Effect of STZ, CTS-SeNPs, or Glib on serum glucose, insulin, and IGF-1 levels

	**Glucose (mg/dl)**	**Insulin (ng/mL)**	**IGF-1 (Pg/mg protein)**
Control	87. 2 ± 2.7^a^	2.1 ± 0.08^a^	33.3 ± 2.2^a^
CTS-SeNPs	86.3 ± 5.2^a^	2.2 ± 0.17^a^	30.5 ± 4.9^a^
Glib	70.0 ± 4.2^a^	2.2 ± 0.12^a^	31.2 ± 2.3^a^
STZ	599.0 ± 38^b^	1.4 ± 0.19^b^	11.5 ± 3.5^b^
STZ + CTS-SeNPs	87.0 ± 7.4^a^	1.8 ± 0.23^c^	24.5 ± 11.0^c^
STZ + Glib	167.8 ± 30.4^c^	1.9 ± 0.11^d^	29.8 ± 4.2^d^

Values are expressed as mean ± SD in each group (*n* = 8). Different symbols are significant at *p* ≤ 0.05 (Duncan’s test).

### Effect of STZ, CTS-SeNPs, or Glib on Serum α-Amylase and α-Glucosidase Activities

**Fig. 3:**
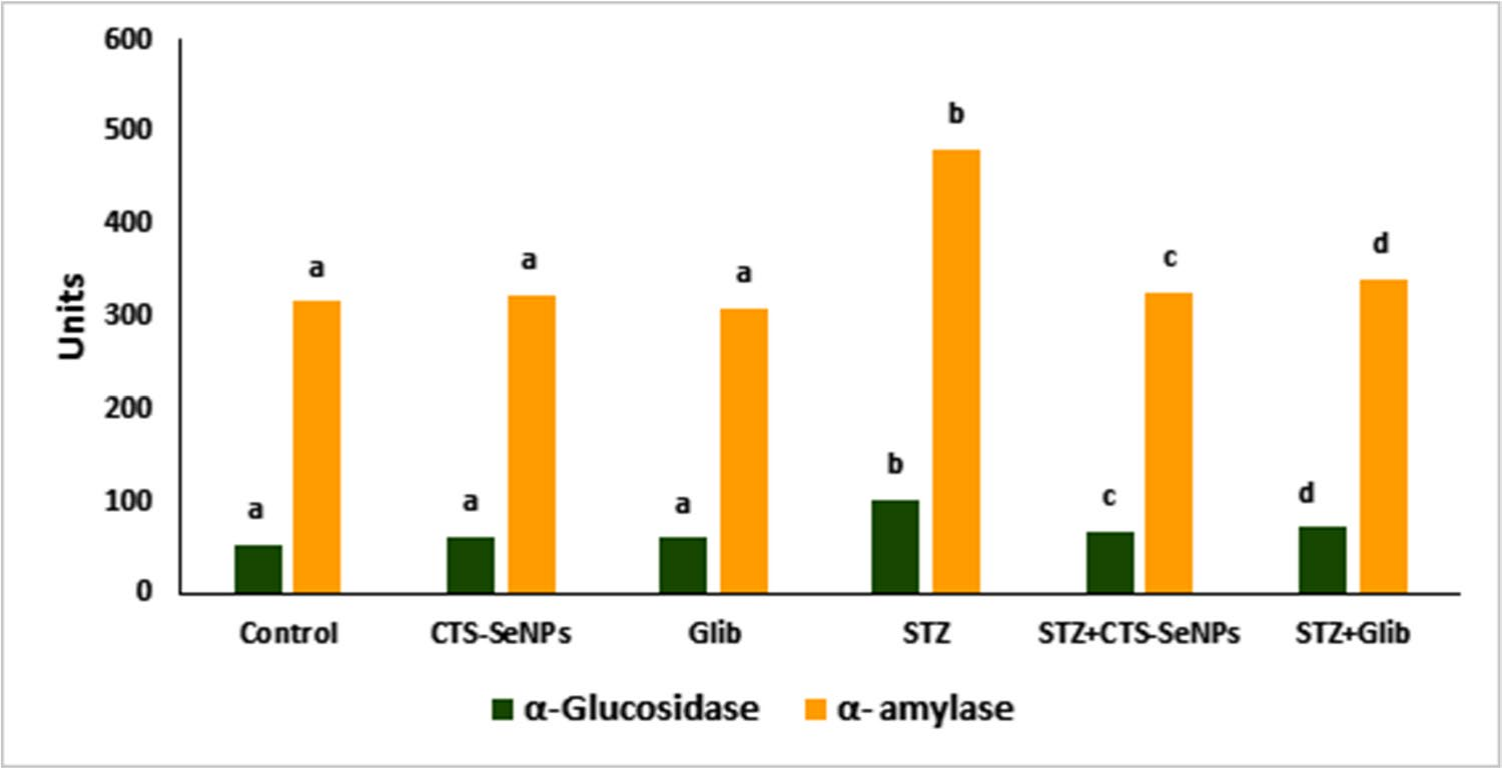
Effect of STZ, CTS-SeNPs, or Glib treatment on serum A: α-glucosidase, B: α-amylase activities in different groups. Values are expressed as mean ± SD in each group (*n* = 8). Different symbols are significant at *p* ≤ 0.05 (Duncan’s test)

Analysis of the data of α-amylase and α-glucosidase activity of diabetic group (STZ) showed a significant (*p* ≤ 0.05) increase in their enzymatic activities (482.5 ± 17.4 and 103.0 ± 3.3, respectively) as compared to normal control group. Diabetic rats treated with CTS-SeNPs or Glib revealed a significant decrease in α-amylase and α-glucosidase activities (*p* ≤ 0.05) relevant to STZ group (Fig. 3).

### Effects of CTS-SeNPs on Body Weight in CTS-SeNPs-Fed Rats

**Fig. 4:**
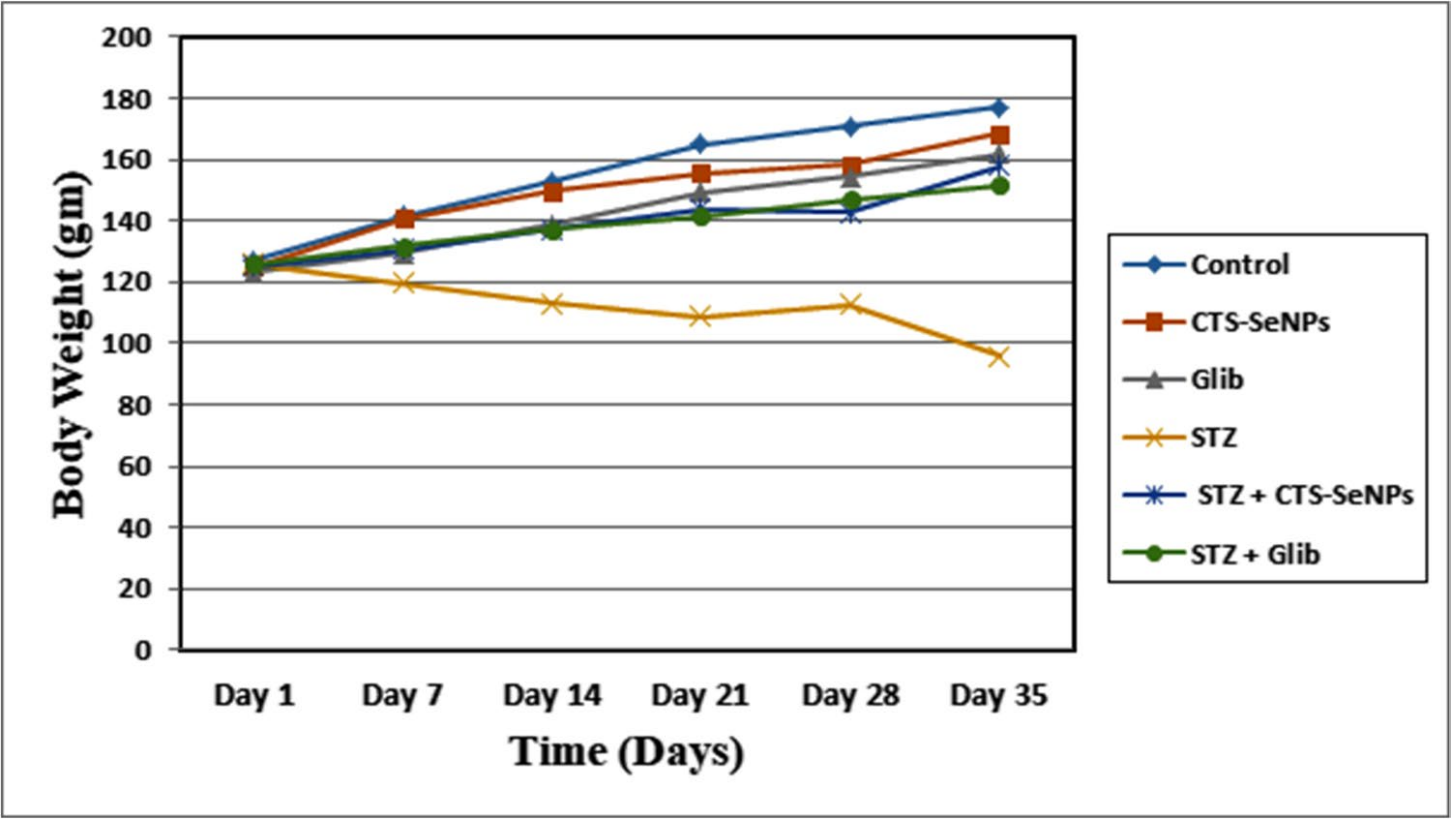
Effect of STZ, CTS-SeNPs, or Glib treatment on body weight in different animal groups. Values are expressed as mean ± SD

Results presented in Fig. 4 of BW showed insignificant changes (*p* > 0.05) in BW levels of CTS-SeNPs and Glib groups compared to control group. Treating male rates with STZ showed significant decrease in BW level compared to control group. On the other hand, treating diabetic rats with CTS-SeNPs or Glib showed significant increase (*p* ≤ 0.05) in BW in STZ + CTS-SeNPs and STZ + Glib groups compared to STZ group**.**

### Effect of STZ, CTS-SeNPs, or Glib on Serum Antioxidant and Oxidative Stress Markers

**Fig. 5:**
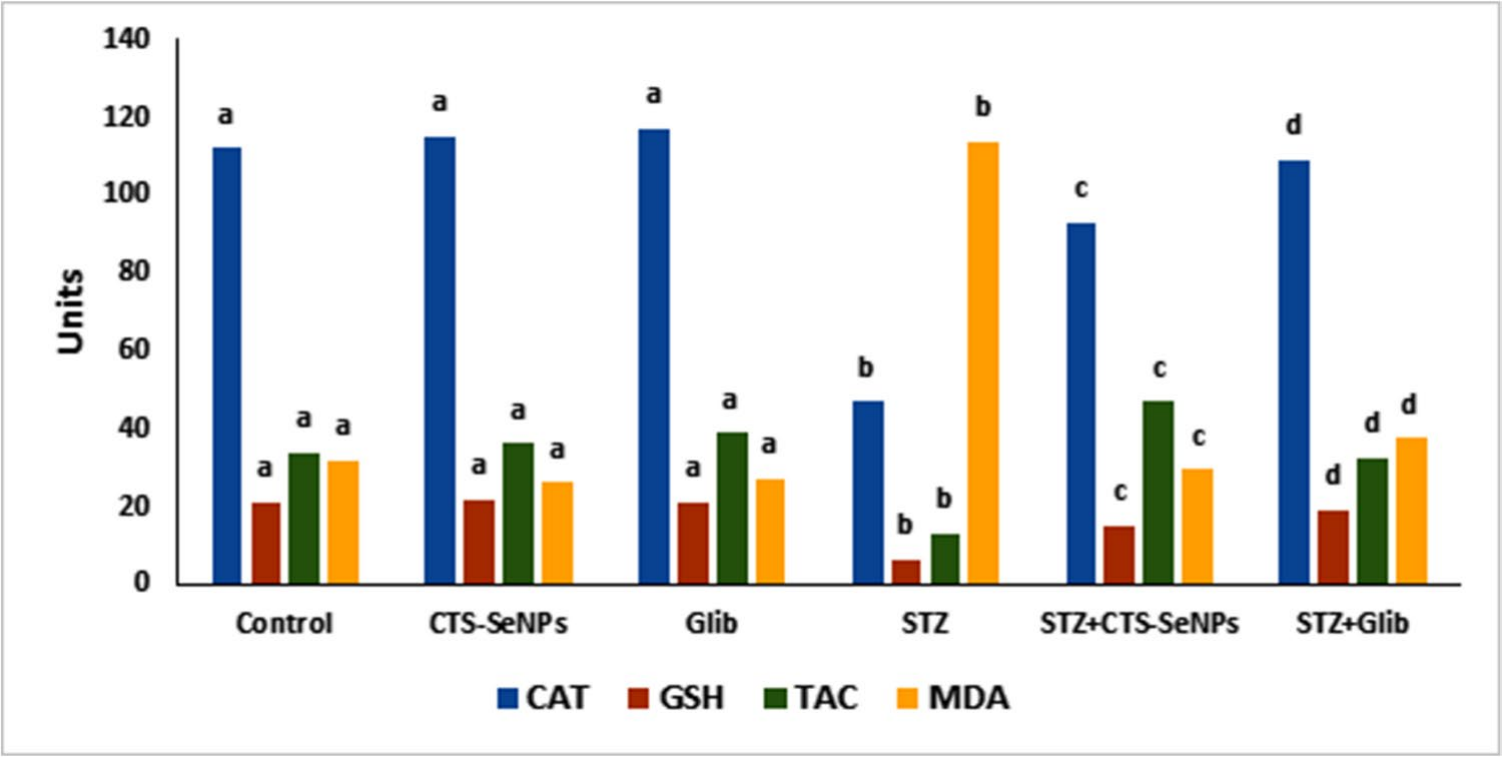
Changes in serum CAT, GSH, TAC, and MDA levels among different groups after treatment with STZ, CTS-SeNPS, or Glib. Values are expressed as mean ± SD in each group (*n* = 8). Different symbols are significant at *p* ≤ 0.05 (Duncan’s test)

Treating normal rats with STZ induced an oxidative stress as revealed from the massive reduction in the levels of serum GSH, TAC, and CAT activity with the significant augmentation in MDA level, compared to their normal counterparts. On the opposite side, administration of CTS-SeNPs or Glib to diabetic rats ameliorated the above parameters, compared to STZ group (Fig. 5).

### Effect of STZ, CTS-SeNPs, or Glib on Proinflammatory Markers

**Fig. 6:**
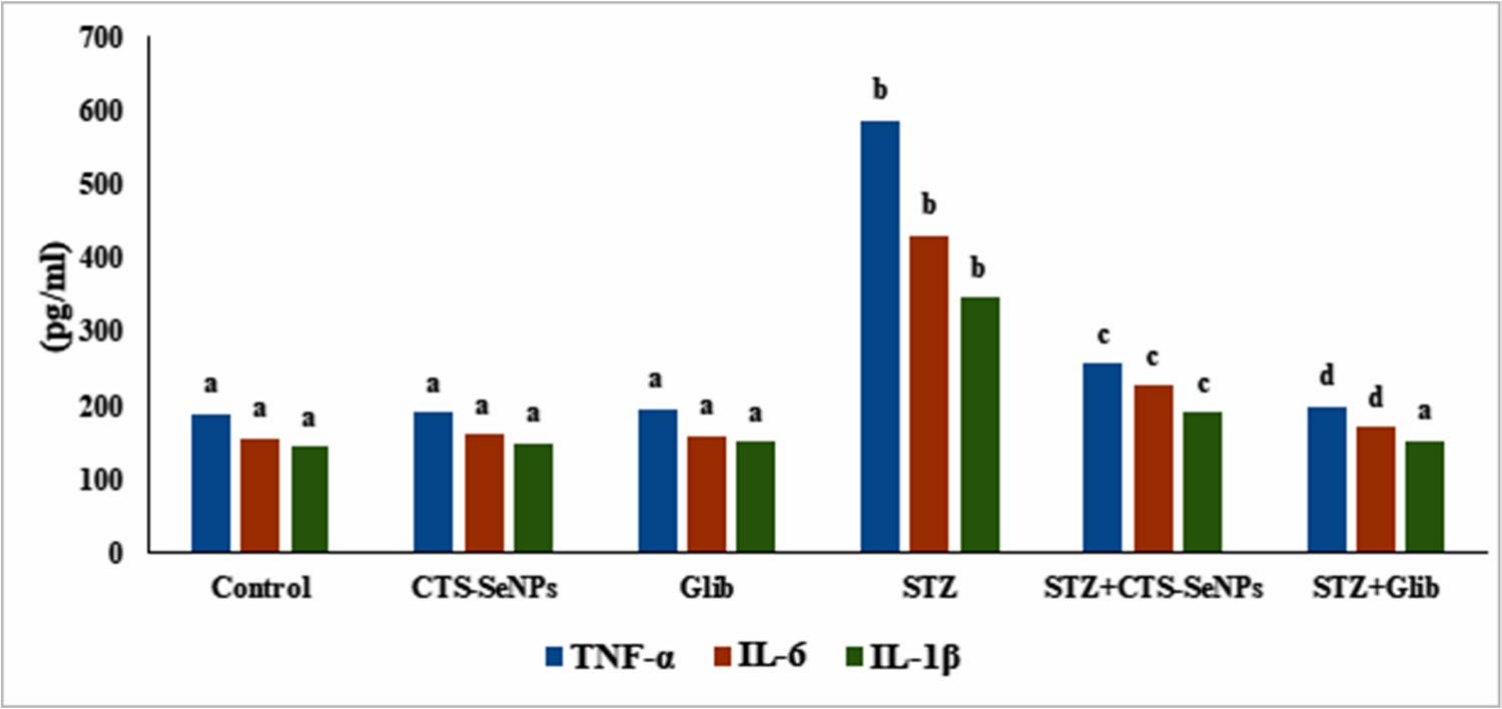
STZ, CTS-SeNPs, or Glib post treatment changes in serum TNF-α, IL-6, and IL-1β levels among different groups. Values are expressed as mean ± SD in each group (*n* = 8). Different symbols are significant at *p* ≤ 0.05 (Duncan’s test)

The levels of serum proinflammatory parameters (TNF-α, IL-6, and IL-1β) in control and treated animals are illustrated in Fig. 6. Treating normal animals with STZ produced a marked elevation in serum levels of the studied proinflammatory parameters, versus normal control. Administrating CTS-SeNPs or Glib to diabetic rats significantly reduced serum levels of the proinflammatory parameters.

### Effect of STZ, CTS-SeNPs, or Glib on Serum Liver and Cardiac Enzymes

**Fig. 7:**
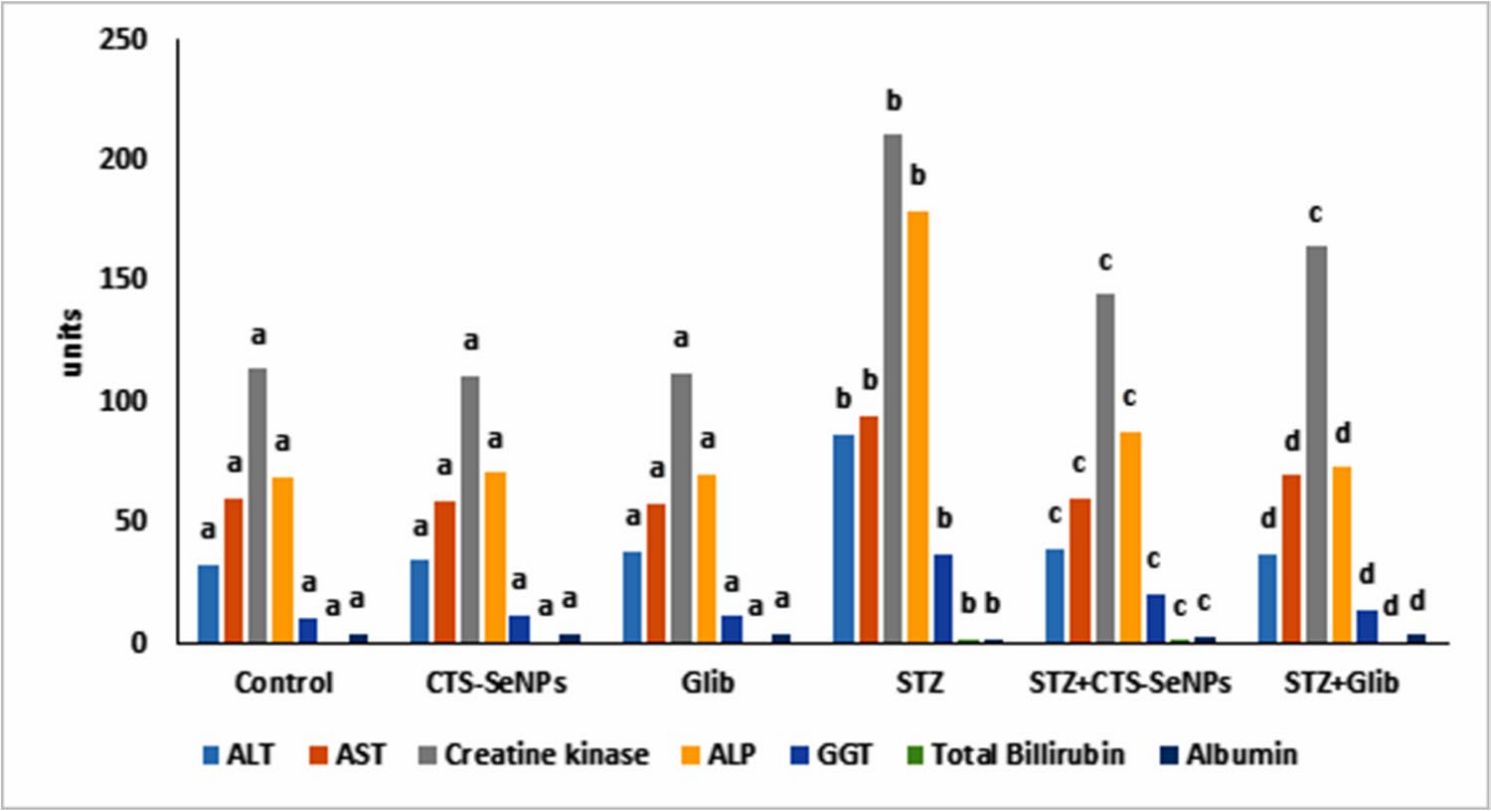
Effect of STZ, CTS-SeNPs, or Glib on serum ALT, AST, ALP, GGT, total bilirubin, albumin, and CK-MB. Values are expressed as mean ± SD in each group (*n* = 8). Different symbols are significant at *p* ≤ 0.05 (Duncan’s test)

Intraperitoneal injection of normal animals with STZ significantly elevated serum hepatic and cardiac enzymes (ALT, AST, ALP, GGT, total bilirubin, albumin, and CK-MB), compared to normal group. Administrating CTS-SeNPs or Glib to diabetic rats ameliorated the above enzymes (Fig. 7).

### Effect of STZ, CTS-SeNPs, or Glib on Serum Lipid Profile

Table 4 revealed that administration of STZ to normal rats induced a marked elevation in the levels of serum total cholesterol, TG, and LDL-C with a significant reduction in the level of HDL-C, as compared with normal group. Treating diabetic animals with CTS-SeNPs or Glib ameliorated the aforementioned parameters.

**Table 4:** Effect of STZ, CTS-SeNPs, or Glib on serum lipid profile

	**Total cholesterol (mg/dl)**	**Triacylglycerol mg/dl)**	**HDL-C (mg/dl)**	**LDL-C (mg/dl)**
Control	104.5 ± 3.6^b^	67.5 ± 2.7^b^	38.7 ± 1.2^b^	59.0 ± 2.4^b^
CTS-SeNPs	102.3 ± 4.6^b^	67.8 ± 4.5^b^	39.0 ± 1.3^b^	55.3 ± 3.8^b^
Glib	88.5 ± 39^b^	67.5 ± 5.4^b^	38.1 ± 1.5^b^	49.2 ± 3.1^a,b^
STZ	206.5 ± 11.1^a^	179.0 ± 5.3^a^	18.2 ± 0.98^a^	157.7 ± 9.2^a^
STZ + CTS-SeNPs	102.7 ± 4.2^b^	66.3 ± 3.0^b^	37.8 ± 1.9^b^	56.0 ± 4.2^b^
STZ + Glib	104.7 ± 7.5^b^	63.8 ± 3.2^b^	38.3 ± 1.5^b^	55.2 ± 5.6^b^

Values are expressed as mean ± SD in each group (*n* = 8). Different symbols are significant at *p* ≤ 0.05 (Duncan’s test).

### Quantitative Real-Time PCR Analysis of TC7L2, CAPN10, and PPAR-γ

**Fig. 8:**
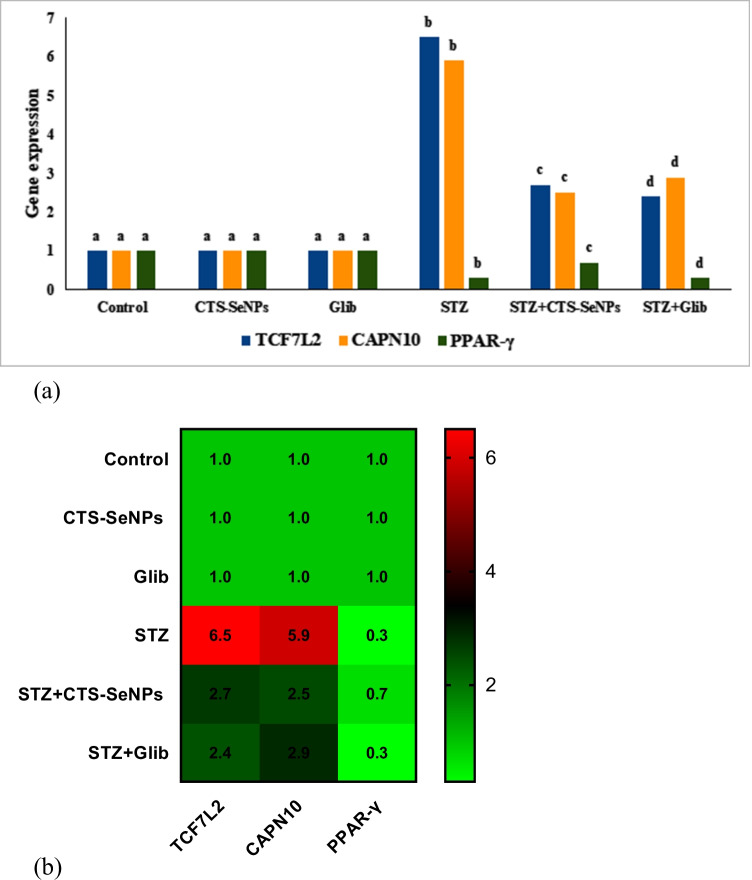
Changes in pancreatic TC7L2, PPAR-γ, and CAPN10 gene expression levels among different groups. Values are expressed as mean ± SD in each group (*n* = 8). Different symbols are significant at *p* ≤ 0.05 (Duncan’s test). b Heatmap diagrams for changes in pancreatic TC7L2, PPAR-γ, and CAPN10 gene expression levels among different groups. The red color indicates the higher fold change gene expression, and the green color refers to the lower ones

The expression levels of TC7L2 and CAPN10 genes in the pancreatic cells of STZ treated rats were significantly increased (*p* ≤ 0.05), compared to control group. On the other hand, STZ-treated animals exhibited a marked downregulation in PPAR-γ gene, compared to their normal counterparts. Treating diabetic rats with either CTS-SeNPs or Glib led to downregulation of both TC7L2 and CAPN10 genes with an upregulation of PPAR-γ gene compared to STZ-treated rats (Fig. 8a and b).

### Histopathological Findings

**Fig. 9:**
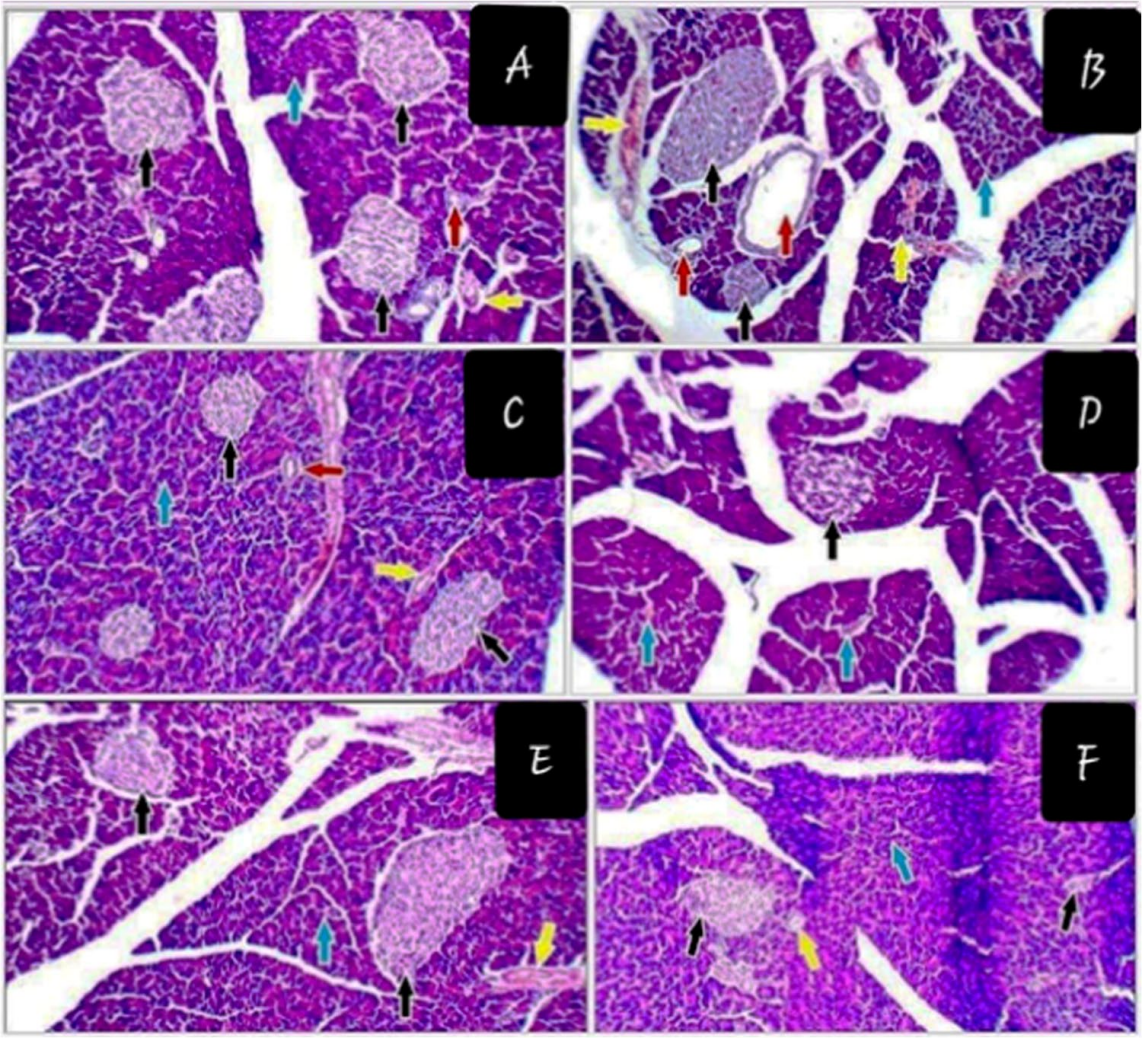
Histopathological photographs of pancreatic tissue: (**A**) normal control, (**B**) CTS-SeNPs, (**C**) Glib, (**D**) STZ, (**E**) STZ + CTS-SeNPs, and (**F**) STZ + Glib. Arrows representing average-sized pale-staining islets of Langerhans (black arrows), exocrine areas (blue arrows), average ducts (red arrow), and interstitial blood vessels (yellow arrow) (H&E × 200)

Histopathological investigation of pancreatic tissue under the light microscope of (A) control animals, (B) CTS-SeNPs, and (C) Glib groups revealed normal texture pattern with contact islets of Langerhans and normal thickness septa, respectively. In STZ group (Fig. 9D), septa was wider than the remaining groups, the islets of Langerhans showed signs of atrophy and shrinking. Infiltrated lymphocytes were seen which indicates inflammation of these cells. Diabetic animal groups treated with CTS-SeNPs or Glib revealed restoration of normal shape and size of pancreatic cells (Fig. 9E and F), respectively. The score for the histopathological investigation of different parts of pancreatic tissue is illustrated in Table 5.

**Table 5:** Histopathological results of pancreatic tissue

Groups	Pancreatic islets					Exocrine area	Ducts	BV
	Islet size	Cellularity	Beta cells	Edema	Capillaries			
A	0	0	0	0	0	0	0	0
B	0	0	0	0	+ +	0	+	+ +
C	0	0	0	0	0	0	0	0
D	+	+	+	+	+	0	0	+
E	0	0	0	0	0	0	0	0
F	0	+	0	0	0	0	0	0

## Discussion

Diabetes mellitus is considered a major global health issue that has reached alarming levels. It is a cluster of several chronic lifelong metabolic endocrine disorders with long-term damage, dysfunction, and failure of various organs, especially the eyes, kidneys, nerves, heart, and blood vessels [24, 25]. Epidemiological studies reveal that the incidence of diabetes is on the rise around the world, with an estimation of approximately 387 million people. This figure is predicted to rise to 590 million by 2035 [26].

Our results demonstrated a significant decrease in serum IGF-1 levels in STZ treated animals compared to the control group. Rajpathak et al., Chu et al., and Zhang et al. [39–41] recorded the association of the reduction in IGF-1 level with the decrease in insulin concentration in diabetes mellitus.

According to Ramesh [27], the main causes of the pathogenesis of diabetes are elevated oxidative stress and mitochondrial dysfunction. Diabetes mellitus is characterized by hyperglycemia associated with increased formation of ROS, reactive nitrogen species, advanced glycation end products, elevated lipolysis, ketogenesis, and diminished levels of antioxidants. Baynes [28] proved that uncontrolled ROS production frequently causes damage to cellular macromolecules (DNA, lipids, and proteins), contributing to the progression of diabetic complications and different organ damage. The generation of ROS due to hyperglycemia stimulates apoptosis, further leading to diabetic microvascular complications. So, it is necessary to investigate new clinical therapies against oxidative stress as additional standard treatments for diabetic patients [29].

In the present work, the potential antidiabetic and antioxidant effects of SeNPs capped with chitosan (CTS-SeNPs) as natural products were investigated in an STZ-induced diabetic model. Glibenclamide (Glib) was used as a reference pharmaceutical product in the treatment of diabetes. Chitosan is an attractive agent for drug development given its function in the gastrointestinal tract and its intrinsic safety when taken orally, as it improves solubility, escalates cellular uptake, and allows sustainable delivery of drugs [30]. It has also been confirmed that CTS could be a good stabilizer for selenium nanoparticles [31]. Moreover, Luo et al. [32] reported that selenite-loaded chitosan nanoparticles possess strong antioxidant activity relative to pure selenite. Also, it has been demonstrated that CTS modifies the size, morphology, and stability of SeNPs in liquid dispersions [33]. Encapsulation of selenite into CTS significantly improves the antioxidant properties and promotes a high retention of selenium in cells [34]. The characterization analyses confirmed the spherical shape of the synthesized CTS-SeNPs with a size ranging from 50 to 130 nm. Nearly similar results were reported by Bai et al [34]. Also, FTIR spectrum analysis of CTS-SeNPs revealed three strong peaks for hydroxyl group (-OH), amid I of proteins, and carbon with –H or –N. These findings coincide with Wu et al [35] who reported that FTIR analysis of SeNPs produced using CTS represented peaks observed at 3449, 1648, and 1030 cm−1 regarded as O–H or N–H stretch.

In the present research, STZ (50mg/kg. b.wt) was intraperitoneally administered to normal rats to induce diabetes mellitus. This was revealed by the elevation of serum glucose levels and the reduction in serum insulin levels. Robertson [36] reported that diabetes mellitus comprises a group of chronic diseases characterized by hyperglycemia or diminished insulin secretion or both. The decrease in serum insulin levels in animal models after STZ induction is used as a sign of the inducement of diabetes [37]. Streptozotocin action in β-cells is accompanied by abnormalities in β-cell function. STZ inhibits glucose oxidation and reduces insulin synthesis [38]. Pancreatic β-cell toxicity and diabetic conditions resulting from STZ induction are related to the glucose moiety in its chemical structure, which enables STZ to enter the β-cells via the low affinity GLUT-2 transporter in the plasma membrane [39], because the β-cells of the pancreas are more active than other cells in taking up glucose and so are more sensitive than other cells to STZ challenge.

IGF-1 is a peptide hormone that shares amino acid sequence homology with insulin. It has an insulin-like activity [40]. In addition, IGF-I may have beneficial effects on systemic inflammation, a diabetic risk factor, and on the mass and function of pancreatic β-cells. Its administration to diabetes patients improved their insulin sensitivity [41]. Our results demonstrated a significant decrease in serum IGF-1 levels in STZ-treated animals compared to the control group. Rajpathak et al., Chu et al., and Zhang et al. [41–43] recorded the association of the reduction in IGF-1 level with the decrease in insulin concentration in diabetes mellitus.

In the current work, STZ-induced diabetic rats were treated with the CTS-SeNPs, causing a marked decrease in glucose levels with an increase in insulin and IGF-1 levels. Selenium is an insulin mimetic, which explains its ability to reduce the levels of glucose [44]. Our findings are comparable to those of Vural et al. [45], who observed a reduction in the serum glucose levels of diabetic rats after treatment with sodium selenite. It has been reported that selenate enhanced the transportation and uptake of glucose in adipocytes of rats by translocating glucose transporters, such as GLUT-1 and GLUT-2, to the surface of many membranes [46]. Furthermore, the glucose lowering effect of Se might be supported by other mechanisms, such as an acceleration of kidney glucose excretion in rats or stimulation of adipogenesis in adipocytes via stimulating serine/threonine kinases, including the p70 S6 kinase [47]. Also, Guo et al. [48] reported that chitosan improves blood glucose levels and exerts its antidiabetic effect by upregulating the expression of glucose transporters.

The proposed mechanism of Se enhancing insulin action includes activation of insulin receptor sites, serving as cofactors or components for enzyme systems involved in glucose metabolism [49]. Also, Bai et al. [50] reported that selenium’s antidiabetic action results from its behavior as an antioxidant nutrient, since insulin signaling and secretion are associated with the cellular redox state [51]. Similarly, Campbell et al. [52] found that selenium participates in insulin’s action on β-pancreatic cells through its ability to regulate the gene expression of insulin promoter factor 1 and to increase the mRNA expression of insulin in the mouse β-cell of Min6 cells.

The increase of IGF-1 in the diabetic group treated with SNPs may be connected to the earlier reported decrease in both inflammatory activity and oxidative stress, which was caused by supplemental selenium [53].

In our results, Glib administration in diabetic rats showed a significant decrease in glucose and an increase in insulin and IGF-1 levels. The mode of action of Glib in hyperglycemic conditions is to lower blood glucose via stimulating insulin production from the existing β-cells of the pancreas by inhibiting ATP-sensitive potassium channels in pancreatic β-cells. Glibenclamide This inhibition causes the cell membrane to increase insulin secretion due to the closure of potassium-ATP channels, opening voltage-dependent calcium channel. It also improves glucose utilization and, as a result, insulin release [54].

α-Amylase and α-Glucosidase are carbohydrate-metabolizing enzymes involved in the breakdown of long chain carbohydrates, starch, and disaccharides to glucose, respectively [55]. Our findings represented elevated α-glucosidase and α-amylase activities in STZ-treated rats. On the other hand, administration of CTS-SeNPs or Glib to diabetic rats significantly reduced α-amylase and α-glucosidase activities. Inhibition of α-amylase and α-glucosidase therefore slows the release of absorbable monosaccharides from dietary complex carbohydrates, postpones the absorption of glucose into the blood, and thus avoids any sudden increase in the amount of blood glucose post meals [56]. Chitosan-SeNPs have been shown to lower serum glucose concentrations by inhibiting the activity of α-amylase and α-glucosidase [57]. Furthermore, Guo et al. [48] reported that chitosan exerts its antidiabetic effect by inhibiting the expression of intestinal α-amylase and α-glucosidase. Moreover, McCue et al. [58] indicated that delaying carbohydrate absorption with Glib offers a prospective therapeutic approach for management of α-amylase and α-glucosidase activities in DM and may be beneficial for borderline diabetic patients.

Our results showed a significant decrease in the body weight of diabetic rats. This could be attributed to the increased catabolic reactions due to the inability to utilize carbohydrates as an energy source, leading to muscle waste [59]. Treating diabetic rats with CTS-SeNPs or Glib restores body weight through the experimental period. This could be due to a post-treatment increase in insulin levels that improved glycemic control, thereby preventing weight loss [60]. Kumar et al. [61] observed that SeNPs had a great effect on restoring body weight. Furthermore, SeNPs had dramatically higher effects on increasing the body weight of diabetic rat. Using chitosan may also improve SeNPs’ effects. Also, Obafemi et al. [62] confirmed that treatment with Glib improves body weight significantly and prevents muscle wasting due to hyperglycemic conditions.

Hyperglycemia causes oxidative stress due to increased mitochondrial production of the superoxide anion, non-enzymatic glycation of protein, and glucose autoxidation. Oxidative stress occurs as a result of either overproduction of ROS or insufficiency of antioxidant defense systems, thus disrupting redox signaling, and is, therefore, implicated in the pathogenesis of major human diseases [63].

The STZ injection induced a state of oxidative stress manifested by elevation of lipid peroxidation and depletion of the enzymatic and non-enzymatic antioxidants in the diabetic rats versus the normal group. In comparison, administration of CTS-SeNPs or Glib in diabetics enhanced the levels of GSH, TAC, and CAT activity. It also ameliorated the levels of MDA, compared to diabetic animals. SeNPs have antioxidant properties along with stimulating antioxidant enzymes that induced scavenging various peroxides. That protects membrane lipids and cellular macromolecules from oxidative injury, decreases MDA concentration, and increases TAC [47]. In addition, CTS plays a role in the induction of free radical scavenging and anti-oxidative enzyme production to suppress oxidative stress by inducing anti-oxidative enzymes and acting as ROS scavengers [64].

A study conducted by Obi et al. [65] clarified that glib has some protective role against oxidative damage in hyperglycemic conditions. An earlier study also agrees with our results, showing that the STZ diabetic rats treated with glib showed a significant reduction in MDA [66].

The liver plays a very important role in the maintenance of glucose levels in the body. It regulates the metabolism of glucose by using glucose molecules as an energy source. Diabetes mellitus may lead to liver-associated diseases such as non-alcoholic fatty liver disease (NAFLD) [67, 68]. ALT, AST, ALP, GGT, total bilirubin, and albumin are considered markers of liver toxicity. According to our observations, STZ-treated animals showed an increase in serum levels of ALT, AST, ALP, GGT, total bilirubin, and albumin. Our results are in agreement with the findings of Ghimire et al. [69], who showed that ALT, AST, ALP, GGT, total bilirubin, and albumin levels were significantly increased in the liver of STZ-treated animals with insulin deficiency, and these changes can be associated with the increase in gluconeogenesis and ketogenesis during diabetes. Treating diabetic rats with either CTS-SeNPs or Glib restored the activities of ALT, AST, ALP, GGT, total bilirubin, and albumin markers to normal values. Al-Quraishy et al. [47] suggested that SeNPs ameliorated STZ dysfunction through radical scavenging activity, besides its integrity and functions of liver tissues resulting from the role of SeNPs in treating liver dysfunction. Moreover, the hepatoprotective effects of chitosan against hepatotoxicity may also have contributed to its anti-inflammatory effect, as shown by reduction of liver enzyme levels [70]. The effects of Glib against diabetes-associated liver injury were confirmed by the decrease in the serum activities of AST and ALT. This effect was concomitant with the improved glucose level [71].

Cardiovascular complications caused by uncontrolled hyperglycemia are widely regarded as one of the leading causes of morbidity and mortality in diabetic patients [72]. Oxidative stress and inflammation have been implicated in playing a central role in the progression of dilated cardiomyopathy (DCM) [73]. Actually, CK-MB is used as a marker of myocardial injury [74]. The current study presented an increase in the circulating values of CK-MB in diabetic rats. These findings were supported by [75]. Treating diabetic rats with CTS-SeNPs significantly lowered CKMB serum levels. Selenium suppresses cardiomyocyte apoptosis through inhibiting the p38MAPK/CBP pathway [76]. Furthermore, selenium restores depressed β-adrenergic responses of the heart in diabetic rats [77]. Besides its antioxidant, hypolipidemic effects [45], and anti-inflammatory activity, it has a beneficial effect on the regulation of the leukotriene pathway in diabetic cardiac hypertrophy hearts [78]. Also, Sutthasupha and Lungkaphin [64] reported the ameliorative effect of chitosan on diabetic cardiomyopathy.

In the setting of diabetes, some sulfonylureas like Glib have been found to inhibit the activity of the ATP-sensitive potassium channels (KATP), resulting in ischemic preconditioning inhibition [79]. In our study, results showed a significant decrease in CK-MB activities in diabetic rats treated with Glib. Glib has more affinity for cardiac monocytes [80]. This provides an explanation for the negative cardiovascular outcomes seen with Glib [79].

Abnormalities in lipid profiles are one of the most common complications in diabetes mellitus, found in 40% of diabetic cases [81]. Our results showed a marked increase in cholesterol, triacylglycerol, and LDL-c with a significant decrease in HDL-c in diabetic rats. Abnormally high concentrations of plasma lipids in diabetes are mainly due to an increase in the mobilization of free fatty acids from the peripheral depots in the absence or deficiency of insulin. Hence, during diabetes, hepatic lipogenesis is decreased, and lipolysis is increased [82]. Insulin deficiency or resistance may be responsible for dyslipidemia, because insulin has an inhibitory action on 3-hydroxy-3-methylglutaryl coenzyme A (HMG-COA) reductase, a key enzyme which is responsible for the metabolism of LDL-c particles rich in cholesterol [83]. Moreover, altered HDL composition in patients with diabetes results in a diminished ability to promote reverse cholesterol transport. Impeded cholesterol efflux from adipose and hepatic cells is mainly related to increased triacylglycerol and decreased cholesterol content of HDL [84]. Jiang et al. [85] demonstrated that the oral treatment of SeNPs results in a remarkable decrease in serum levels of cholesterol, triacylglycerol, and HDL-c which is linked with a significant decrease in LDL-c levels compared to the untreated diabetic rats. The increased HDL-c levels could raise the efflux of cholesterol and triacylglycerol from liver tissue for catabolism. This would result in a reduction in cholesterol triacylglycerol in the blood. These findings are in agreement with our observations.

One of the genes identified in relation to diabetes risk is the transcription factor 7-like 2 gene (TCF7L2). It is the major transcription factor and one of the components of the canonical WNT signaling pathway and is mainly expressed in human pancreatic beta cells and adipose tissue [86]. TCF7L2 has an important role in glucose homeostasis, the generation of insulin resistance, and lipid metabolism [87]. High expression of this gene results in impaired glucose-stimulated insulin secretion [88]. Low insulin secretion and the risk of DM are associated with TCF7L2 because it is a suppressor of GLP-1 in the intestines [89]. Moreover, TCF7L2 functions as a co-activator of p65 to potentiate inflammatory cytokine production in macrophages to aggravate induced chronic inflammation and insulin resistance in mice. Our results indicated that STZ treatment induced an upregulation of the aforementioned gene with the elevation of the inflammatory markers (TNF-α, IL-1β, and IL-6), confirming the incidence of DM and inflammation.

Treating of diabetic rats with CTS-SeNPs or Glib resulted in the downregulation of TCF7L2 gene expression with a reduction in the levels of inflammatory markers, compared to the diabetic group. This coincides with that reported by [90–92].

Calpain-10 (CAPN10) is a Ca^2+^-dependent cysteine protease that regulates glucose homeostasis in pancreatic islet cells, liver, skeletal muscle, and adipocytes [93]. It has an essential function in proinsulin processing and insulin production and activity [94]. This study showed that CAPN10 gene expression was upregulated in STZ-treated rats. Oxidative stress increases intracellular free Ca^2+^ levels and activates Ca^2+^-dependent enzymes which mediate apoptosis [95–97]. In our study, treatment with CTS-SeNPs or Glib resulted in a downregulation of CAPN10 gene expression compared to the diabetic group. SeNPs were found in intronic regions, where they altered gene expression of CAPN10 alternative splicing mechanisms [98].

Peroxisome proliferator-activated receptors-γ (PPARγ) are glycolysis proteins that maintain glucose homeostasis in the liver and pancreas by activating glucokinase and glucose transporter 2 (GLUT2). This gene is associated with postprandial hypertriglyceridemia and plays a major role in the development of diabetes mellitus [99].

In the present investigation, STZ administration resulted in a significant downregulation in (PPAR-γ) gene expression compared with normal nondiabetic animals. CTS-SeNPs or Glib treatment, on the other hand, significantly increased PPAR-γ mRNA expression. Studies have documented that activation of PPAR regulates various genes involved in cell growth and differentiation, inflammatory pathways, insulin sensitivity, angiogenesis, lipid, and glucose metabolism [100]. Peroxisome proliferator-activated receptors-γ have also been shown to inhibit the release of proinflammatory cytokines from macrophages, such as TNF-α and ILs, resulting in pain relief [101]. In contrast, PPAR-γ inhibition activates nuclear factor-kB, resulting in the release of various proinflammatory cytokines (TNF-α and IL-1β), which further activates the pain pathway.

Thus, activation of the PPAR-gene produces an anti-inflammatory effect [101]. The activation of PPAR-γ by CTS-SeNPs treatment is the central mechanism behind the amelioration of diabetic complications and various disease states. Similarly, chitosan exerts its antidiabetic effect by improving the expression of PPARγ [48]. Another study suggested that chitosan prevents hyperglycemia by inhibiting intestinal glucose digestion and helps in transporting and enhancing glucose uptake, at least in part, by upregulating PPARγ expression of adiponectin in adipocytes [48].

Our observations revealed significant upregulation of PPARγ gene expression in diabetic groups treated with Glib. These results indicated strong correlations between PPARγ and the antidiabetic action of sulfonylureas such as Glib. Also, some studies have reported the relative potency of sulfonylureas for binding to PPARγ and activation of PPARγ in vitro, which is well associated with their antidiabetic potency in vivo [102].

The histopathological analysis in the present work goes along with the biochemical and molecular studies. STZ treatment induced toxicity, and damaging signs appeared in the atrophy of the island of Langerhans cells, small-sized pale-staining hypocellular, apoptotic β-cells, predominating alpha cells, and congested blood capillaries. Pisarev et al. [103] concluded that the STZ can cause cellular necrosis and selective destruction of β-cells via its direct alkylating action. On the other hand, CTS-SeNPs-treated diabetic rats showed normal histological structure of the island of Langerhans cells, with average-sized pale-staining islets of Langerhans composed of predominating β-cells. Wang et al. [104] reported that SeNPs limited the dysfunction of β-cells by inhibiting apoptosis and oxidative stress. Notably, chitosan has been shown to promote the proliferation of β-cells and the recovery of damaged β-cell functions, which can produce insulin and thus increase insulin sensitivity [48]. Our results demonstrated that Glib-treated diabetic animals showed regression in cell atrophy caused by STZ with predominating β-cell cytoplasm and newly pancreatic β-cells are present.

## Conclusion

The current investigation demonstrated the therapeutic effect of CTS-SeNPs against experimentally induced diabetes mellitus in adult male rats. This effect is afforded by the antioxidant, hypoglycemic, and hypolipidemic properties of CTS-SeNPs, as a result of using selenium and chitosan. Along with improving vital organ function (pancreas, liver, and heart).

## Data Availability

All the data are available in the current study.
